# Point-of-service, quantitative analysis of ascorbic acid in aqueous humor for evaluating anterior globe integrity

**DOI:** 10.1038/srep16011

**Published:** 2015-11-03

**Authors:** Manas R. Gartia, Santosh K. Misra, Mao Ye, Aaron Schwartz-Duval, Lisa Plucinski, Xiangfei Zhou, David Kellner, Leanne T. Labriola, Dipanjan Pan

**Affiliations:** 1 Department of Bioengineering, University of Illinois at Urbana-Champaign; 2 Beckman Institute of Advanced Science and Technology, University of Illinois at Urbana-Champaign; 3 Department of Materials Science and Engineering, University of Illinois at Urbana-Champaign; 4 Carle Foundation Hospital, 611 West Park Street, Urbana, IL, USA; 5 Department of Electrical and Computer Engineering, University of Illinois at Urbana-Champaign; 6 Research Park, University of Illinois at Urbana Champaign, IL; 7 Department of Surgery, University of Illinois College of Medicine, Urbana IL, USA

## Abstract

Limited training, high cost, and low equipment mobility leads to inaccuracies in decision making and is concerning with serious ocular injuries such as suspected ruptured globe or post-operative infections. Here, we present a novel point-of-service (POS) quantitative ascorbic acid (AA) assay with use of the OcuCheck Biosensor. The present work describes the development and clinical testing of the paper-based biosensor that measures the changes in electrical resistance of the enzyme-plated interdigitated electrodes to quantify the level of AA present in ocular fluid. We have demonstrated the proof-of-concept of the biosensor testing 16 clinical samples collected from aqueous humor of patients undergoing therapeutic anterior chamber paracentesis. Comparing with gold standard colorimetric assay for AA concentration, OcuCheck showed accuracy of >80%, sensitivity of >88% and specificity of >71%. At present, there are no FDA-approved POS tests that can directly measures AA concentration levels in ocular fluid. We envisage that the device can be realized as a handheld, battery powered instrument that will have high impact on glaucoma care and point-of-care diagnostics of penetrating ocular globe injuries.

Eye injuries and ocular complications present to many health care professionals through emergency department visits, convenient care appointments or primary care evaluations; however, accurate ocular examination typically requires specialty training and expert knowledge of the use of ophthalmic diagnostic equipment such as the slit lamp biomicroscope. The limited instruction available on these devices and restricted access to the equipment due to the high cost and immobility, inhibit the ability for primary care providers to adequately diagnose, triage or manage complicated ocular conditions. This is particularly problematic when cases of serious ocular injuries, that require urgent attention, present outside of an ophthalmology office. This occurs with patient with suspected ruptured globe patients or post-operative infections.

Current methods for evaluating the integrity of the anterior globe in trauma patients and the wound integrity in post-operative patients involve the use of the Seidel Test. This test is performed by placing a high concentration of fluorescein dye into the ocular tear film and then observing for a change in the color of the dye. The change in color would indicate the passage of aqueous humor through a corneal or anterior scleral wound, which represents a direct communication of the internal eye fluid with the external tear film. The Seidel Test is subjective and not standardized, and the amount of pressure and technique used when performing this test varies between clinicians^1^. Other devices that are used to aid in diagnosis of trauma patients include conventional X-ray, computed tomography (CT), ultrasound (US) and magnetic resonance imaging (MRI), but they are limited in their capability to detect eye injuries. Specifically, plain film radiographs have no utility in detecting soft tissue injuries to the eye; CT images do not visualize small anterior lacerations to the cornea and US is contraindicated with anterior globe ruptures. In addition, all of these imaging devices are expensive and are restricted to hospital settings due to their size and cost. Furthermore, none of these devices are available for evaluation of an eye trauma by first responders in the field or for military use in combat settings.

In this research, we present a novel method that provides an objective, reliable platform for testing ascorbic acid (AA) within the ocular tear film as a surrogate biomarker of anterior scleral or corneal wound integrity, which could replace the subjective Seidel Test. Our method utilizes the 20-fold difference in AA concentrations found in ocular fluids. Aqueous humor has an average AA concentration of 1049 ± 433 micromol/L^2^,^3^,^4^ whereas the ocular tear film only has an average AA concentration of 23 + 9.6 micromol/L^5^. With this fundamental difference in concentration and the knowledge that aqueous humor is continuously produced within the anterior chamber, we hypothesize that when the integrity of the anterior globe is disturbed from a full-thickness laceration, the higher concentrations of AA from within the continuously flowing aqueous humor will be released into the tear film causing a rise in the amount of AA in the tear film that can be quantified. The tear film AA concentration can be measured by our novel, point-of-service device called the OcuCheck Biosensor™.

There are currently no FDA-approved point-of-service (POS) tests that directly measure AA in tear film. Other methods of AA detection include HPLC^6^,^7^, electrochemical^8^,^9^,^10^,^11^,^12^,^13^, colorimetric^5^,^14^,^15^,^16^,^17^,^18^,^19^,^20^, absorbance and fluorescence measurement but all of them have serious limitations of requirement of sophisticated instrumentation, limitation to low concentration detection and extensive sample preparation (Table S1). Our strategy involves the use of nanotechnology through an enzyme-graphene decorated plated electrode to quantitatively measure the concentration of tear film AA. An important feature of the biosensor that sets it apart from current care options is that the biosensor reports the level of AA concentration on an electronic screen, making the results easy to read and suitable for use by a non-ophthalmologist. Our method of using an electrical resistance based biosensor overcomes other shortcomings of current techniques for AA detections. The resistance-based measurement provided by the OcuCheck can be performed in the clinical setting with an immediate result without having to send the samples to a laboratory for further sampling or analysis, as competing assays require. This feature of the OcuCheck device has proven to be a vital feature to enable clinical use for the device due to the fact that AA rapidly degrades after collection^21^. In previous studies L-ascorbic acid solution degraded during storage for longer period in the presence of oxygen due to oxidation of AA. It involves the loss of two electrons and two protons while oxidation product Dehydroascorbic Acid (C_6_H_6_O_6_) is relatively unstable in aqueous solution, since it spontaneously reacts with water to yield 2,3-diketogulonic acid. The rate of oxidation depends on the concentration of oxygen, temperature, enzyme or transition metal catalysis or basic pH abundance. The ability of the OcuCheck to test the tear film immediately avoids the problems that occur with oxidation and increases accuracy of the test.

The validity of the results obtained from OcuCheck is shown by the strong correlation with the AA concentration obtained through standard colorimetric coupled enzyme reaction assay and mass spectrometric based analytical methods. The OcuCheck provided accurate AA concentration within 5 minutes using 2 μL of sample, suggesting laboratory quality data can be realized with this technique of using a battery-powered, handheld unit with disposable biosensor strips.

## Results

### Design and fabrication of OcuCheck

The unique design of the OcuCheck multilayered test strip utilizes the selective properties of the enzyme ascorbate oxidase (AO) which is placed on graphene platelets and an amphiphilic diblock-copolymer, Poly(styrene)-block-poly(acrylic acid) (PS-b-PAA). Graphene nanoplatelets (STREM CHEMICALS, USA) represent a new class of carbon materials with multifunctional properties having “platelet” morphology. Platelet morphology is defined as having a very thin (~6–8 nm) but wide aspect ratio with width of ~25 μm. This unique morphology makes these particles especially effective at providing barrier properties. In addition, their pure graphitic composition delivers excellent electrical and thermal conducting properties. The paper-based sensor is made by coating the filter paper (Whatman^TM^; GE healthcare, UK limited; 90 mm qualitative circles) with multiple layers of composite containing Poly(styrene)-block-poly(acrylic acid) (PS-*b*-PAA) and graphene platelets. The substrate for the sensor was produced by the sequential deposition of graphene platelet over the filter paper followed by casting a layer of PS-*b*-PAA. The non-covalent π-π stacking interaction between the two dimensional graphene platelets and multiple repeat units of poly(1-phenylethylene) functionalities of the diblock-copolymer was realized.

**Figure 1:**
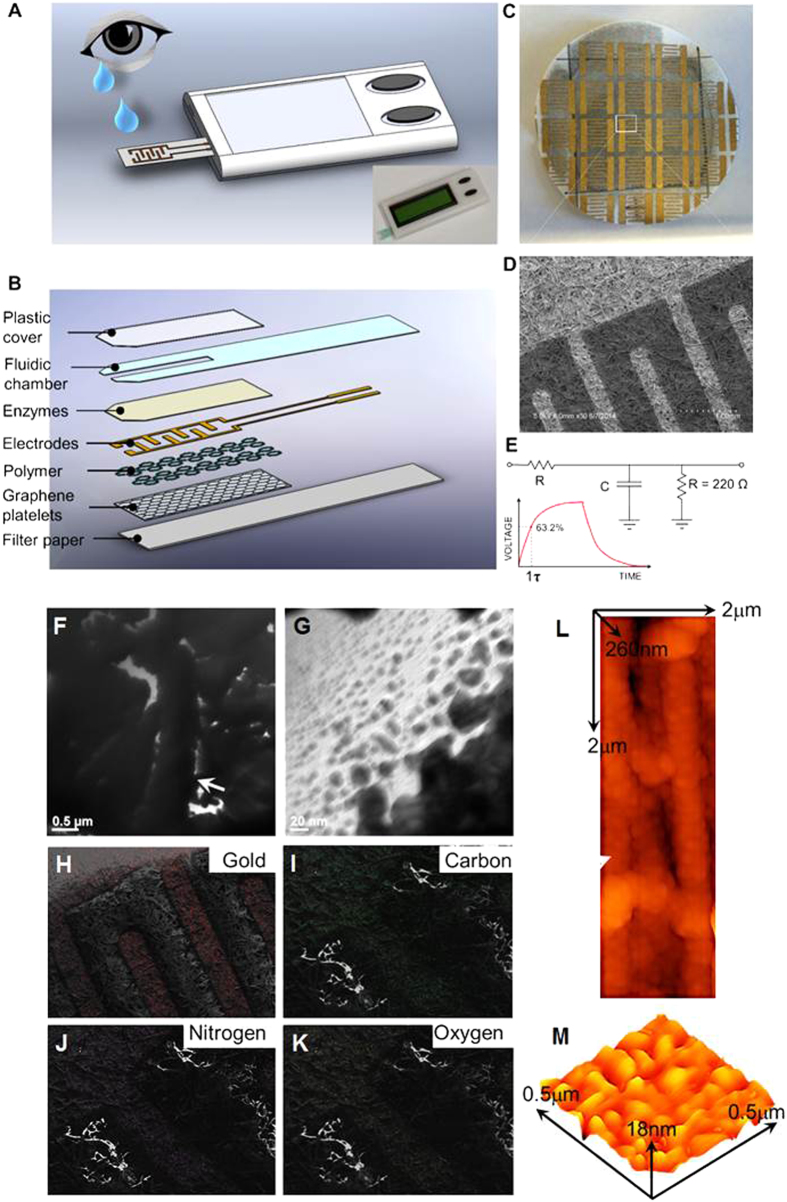
Schematic and operating principle of OcuCheck. (**A**) Graphical schematic of the OcuCheck and sample processing. The inset shows an image of the 3D printed cassette along with LCD screen and the sensor. (**B**) The device consists of interdigitated gold electrodes placed between layers of graphene platelets mixed with polymers, and ascorbate oxidase enzyme. The sensor is laminated with polyester fluidic chamber and a cover. The whole structure is supported on a filter paper. (**C**) Optical image of the sensor made on filter paper and the corresponding SEM image is shown in (**D**). (**E**) The circuit diagram used to measure the surface resistance of the sensor. (Fig. 1 A & B drew by XZ. The schematic of eye in Fig. 1A is taken from http://www.wpclipart.com/people/bodypart/eye/Eye_Tear.png.html and the term of use is provided here http://www.wpclipart.com/terms.html). The graphical representations (**A,B**) of the sensor are 3D rendered in our laboratory. Surface characterization of biosensor strips using (F-G) TEM. Representative TEM show the variation in (**F**) high coating and (**G**) low coating biosensor strips. (**H,K**) SEM/EDX analysis for the elemental map of biosensor chip for elements (H) gold; (**I**) carbon; (**J**) nitrogen and (**K**) oxygen, respectively. AFM analysis shows the representative (**L**) polymer coated fibers on paper sensor and (**M**) height profile of platelets across sensor strip.

The high specificity of the sensor to detect AA is achieved by coating the substrate with the AO enzyme on top of the graphene-polymer coating. The acrylic acid (-CO_2_H) residues of the diblock copolymer were available for facile immobilization of the enzyme over the graphene platelet-polymer composite. We anticipate that the specific interaction of AA with AO will produce a difference in the resistance, which can be measured by an impedance-based detector. The disposable sensor ‘strip’ was designed to measure the AA in a clinical sample by measuring the change in resistance using a handheld multimeter (Fig. 1A). This reading provides a time sensitive result with the final reading appearing in less than five minutes. The inset of Fig. 1A shows the first prototype of the 3D printed model of the handheld OcuCheck biosensor. The layered architecture and components of the sensor are schematically shown in Fig. 1B. The AO layer was immobilized on top of the polymer layer to expose it for binding events with the AA molecules (Fig. S1). In order to measure the changes in the surface resistance of graphene layers due to binding of AA with the oxidase, about 200 nm of gold were deposited on the top of the sensors as interdigitated electrodes format.

The structural integrity of OcuCheck biosensor strip was also established by SEM imaging before and after immobilization of AO enzyme (Fig. 1D). The characteristic surface topography resembling ‘flakes’ type of sensor without AO, (Fig. S2A) was significantly altered to ‘thicker’, denser coating pattern post immobilization with AO (Fig. S2B). A simple RC circuit (Fig. 1E) was used to measure the time it takes for a pin to get charged to a defined voltage value (63.2% of the maximum value), which corresponds to one time constant τ = RC. By connecting a fixed capacitor and a variable resistor in an RC circuit, we used an I/O pin to measure the value of the variable resistor (OcuCheck). The resistance reading was then displayed on the connected LCD monitor screen.

Surface characterization of biosensor strips revealed the variation in properties of strips with high and low density graphene platelet surface coatings and presence and absence of ascorbate oxidase to some extent. A clear difference in pattern was visible in representative areas using TEM (Fig. 1F–G). The study on elemental analysis with SEM/EDX (Fig. 1H–K) of biosensor strips shows abundance of gold (Fig. 1H), carbon (Fig. 1I), nitrogen (Fig. 1J) and oxygen (Fig. K). AFM analysis revealed the presence of polymer coated fibers on paper sensor (L) and height profile of platelets across sensor strip (M).

### Computational studies regarding specificity of the sensor

**Figure 2:**
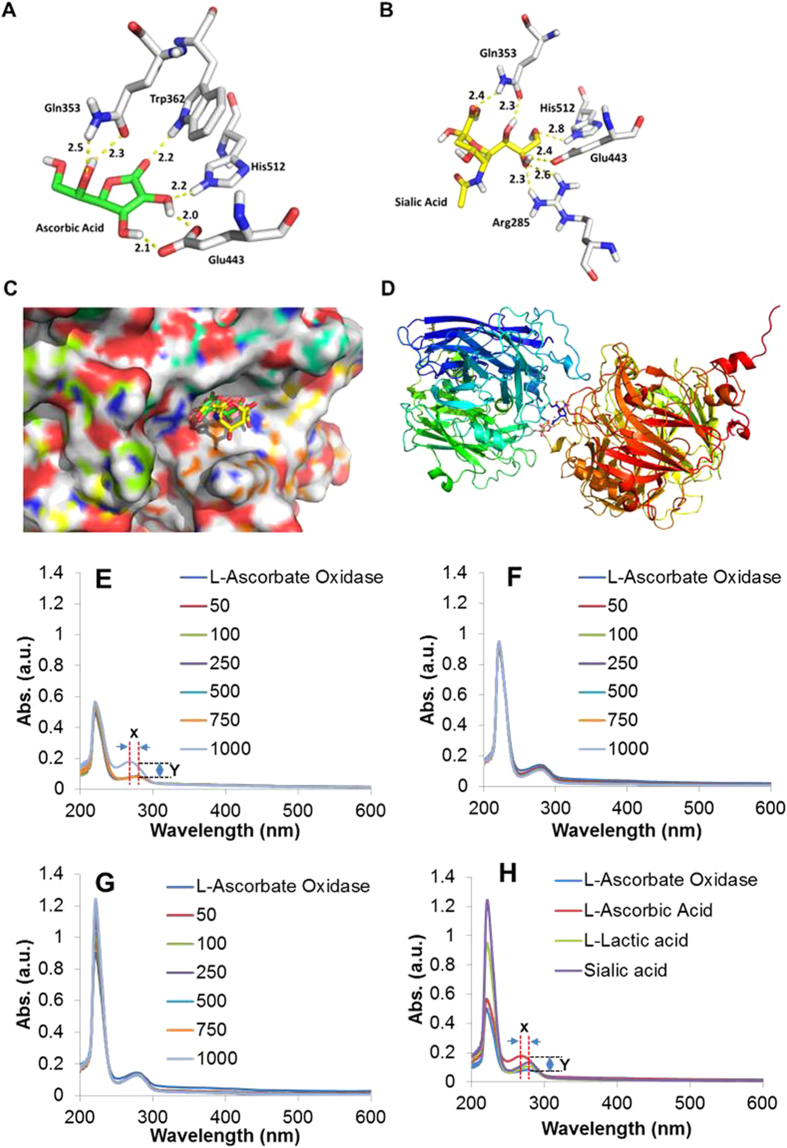
Computational and experimental study of binding affinity and selectivity of ascorbic acid (AA) to the enzyme coating the sensor . (**A**) Docking pose of AA with 1AOZ. (**B**) Docking pose of sialic acid with 1AOZ. (**C**) Molcad surface picture of superimposition of AA and sialic acid docking poses. (**D**) Image showing AA bound to ascorbate oxidase. Selectivity of Ascorbate oxidase toward ascorbic acid. Interaction study at various concentrations (50-1000 μM) of (**E**) L-ascorbic acid; (**F**) L-lactic acid and (**G**) Sialic acid. (**H**) Comparison of interactions at 1000 μM showed only interactions of L-ascorbate oxidase with L-ascorbic acid with changes in λmax of absorption (**x**) and absorption maxima (**y**) while interactions with L-lactic acid and sialic acid showed no significant interaction.

The human tear film is a complex three-layered structure comprising an outermost lipid layer, an aqueous layer and a mucus gel layer^22^. The lipid layer mainly consist of cholesterol esters, and ester waxes; the aqueous layer mainly contains water (99%), proteins (lysozyme, lactoferrin, lipocalin, secretory IgA), electrolytes (Na^+^, K^+^, Cl^-^, HCO^-^, Mg^2+^, Ca^2+^), and small molecules (ascorbic acid, glucose, lactate, uric acid and sialic acid)^23^,^24^,^25^,^26^,^27^. The main component of the mucus layer is mucin, which in turn is characterized by a polymeric assembly of units forming linear polyanionic molecules. Sialic acid is expressed in human tear film and is partly derived from mucin networks and is believed to provide the viscosity^28^. Interestingly, sialic acid is a structurally similar molecule as compared to AA. To understand the disparity in interactions of sialic acid with AO as compared to that of AA to AO, we performed a computational docking experiment^29^. Molecular docking of AA and sialic acid to the binding pocket of AO (1AOZ) (Fig. 2A–D) revealed the following specific recognition events. First, in the AA docking pose, hydroxyl groups of the furan ring, other than that of the side chain, show more H-bond interactions with residues of the target. Then, in the sialic acid docking pose, there were no H-bond interactions between hydroxyl groups of the pyran ring and residues of the target. Instead, all H-bond interactions are exhibited between hydroxyl groups and carboxylic acid group of the side chain and residues of the target. We concluded that from the H-bond distance difference between the two docking poses (Fig. 2A,B), it can be determined that AA shows stronger H-bond interactions with residues of the target than with sialic acid. This shows that AA can be differentiated compared to sialic acid, which is present within the tear film and concentrations of AA can be accurately recorded without competitive inference from sialic acid. This contributes to the high specificity of the OcuCheck biosensor. This study established the integral specificity of the sensor coated with AO toward AA, minimizing the possibility of false positive results (specificity of 71% and false positive rate of ~6% was obtained in the preliminary testing).

Beyond computational docking studies, specific recognition of ascorbic acid on biosensor strip coated with ascorbate oxidase required the experimental evaluations too. Two other major components of aqueous humor, L-lactic and sialic acids, were chosen as competitors. UV-Vis spectroscopic studies were performed to achieve the selectivity. The integral absorbance of ascorbate oxidase was evaluated from its aqueous solution possessing 60 μg (20 U)/mL originated due to presence of aromatic side chains of some amino acids present in ascorbate oxidase (Fig. 2E–H). It was found that sequential addition of aliquots (1 μL; Final concentration 50 μ) of L-ascorbic acid enhanced the absorbance value (Y) of ascorbate oxidase solution (Fig. 2E,H) with a shift of λ_max_ (x) from 280 nm to 267 nm indicating the loss of some aromaticity which might be occurring due to interaction of ascorbate oxidase with ascorbic acid. On the other hand, absorption spectrum of ascorbate oxidase with L-lactic acid (Fig. 2F) and sialic acid (Fig. 2G) did not reveal any significant change in either absorption level or λ_max_ emphasizing the fact of selective interaction of ascorbate oxidase with L-ascorbic acid.

### Layer by layer assembly of OcuCheck and characterization by Raman spectroscopy

**Figure 3:**
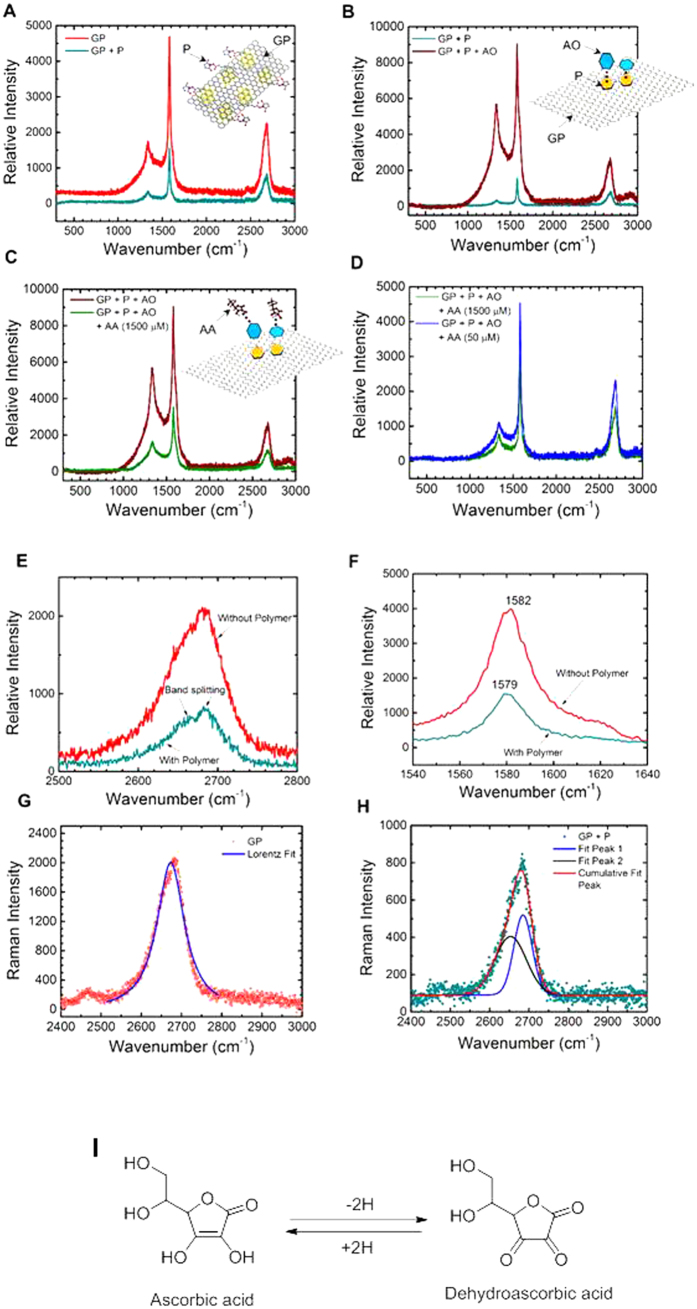
Surface characterization study using Raman spectroscopy to understand the layer-by-layer assembly of OcuCheck and chemistry of enzymatic action . (**A**) Comparison of Raman spectra with and without polymer (P) layer on graphene platelets (GP). (**B**) Comparison of Raman spectra with and without ascorbate oxidase enzyme (AO) layer on polymer coated graphene platelets (GP + P). (**C**) Comparison of Raman spectra with and without ascorbic acid (AA) on polymer coated graphene platelets with enzyme layers (GP + P + AO). (**D**) The effect of concentration of AA on the Raman spectrum of GP + P + AO layers. (**E**) The result showing splitting of 2D-band of graphene after coating the graphene platelets with polymer. (**F**) The G-band of the graphene shifted to lower energy (wavenumber) after coating the graphene platelets with polymer. Results showing the single Lorezian curve fit to data obtained without polymer layer (**G**), and two Lorenzian curves to fit the peak obtained from graphene platelets with polymer layer (**H,I**) Chemistry of ascorbic acid degradation by ascorbate oxidase to generate ehydroascorbic acid.

Raman spectroscopy was used to validate and optimize the layer by layer assembly of OcuCheck biosensor. The Raman spectrum of the sensor surface showed the presence of D, G and 2D peaks (at 1340, 1582, and 2685 cm^−1^, respectively) as clearly visible in Fig. 3A–D. With the subsequent polymer layer on the graphene platelet, the π-π interaction between Graphene-Polymer layers was manifested. Due to additional π-π interaction of graphene and polymer layer compared to graphene layers alone, more energy was required to vibrate the bonds, making it difficult to polarize the system. Therefore, the overall intensity decreases (cyan curve) after the formation of the polymer layer compared to without the polymer layer (red curve) as shown in Fig. 3A. Further evidence of π-π interaction was demonstrated in Fig. 3F. It is well known that the G band position shifts to lower frequency as the number of layers of Graphene increases (Fig. S3B). Figure 3F shows that the G band position is shifted from 1582 to 1579 cm^−1^ due to the polymer layer, similar to the π-π interaction expected from graphene-graphene stacked layer. The 2D band splitting shown in Fig. 3E further confirms the stacking and π-π interaction due to polymer layer (the Lorentz curve fit to the G peak is shown in Fig. 3G,H, corresponding to the curve with and without the grapheme polymer layers, respectively). With the subsequent layering of AO on top of the polymer layer, the Raman spectrum is affected due to interplay between π-π interaction of Graphene-Polymer and Polymer-Oxidase layers. The Polymer-Oxidase interaction lessens the effect of Graphene-Polymer Interaction and hence the overall intensity rises (Fig. 3B: wine red curve) as compared to testing without the oxidase layer (Fig. 3B: cyan curve). Figure 3C shows the result due to interaction between Polymer-Oxidase and Oxidase-AA. The Graphene-Polymer interaction increases again due to Oxidase-AA strong interaction. This leads to overall intensity decrease (Fig. 3C: green curve) after placing AA on the sensor. The hypothesis that interaction of AA-AO leads to decrease of overall intensity of D, G, and 2D band, is tested by placing two different concentrations of AA on the Graphene-polymer-oxidase layer. As expected, an increasing concentration of AA leads to a proportional decrease in the overall intensity of D, G and 2D band (Fig. 3D). The evidence of stacking of layers and π-π interaction is further confirmed by comparing the G band position, full-width-half-maximum (FWHM) of G band, and intensity ratio of D and G band (I_D_/I_G_) after each step (polymer, oxidase, AA, with/without serum) (see Fig. S3A). Polymer layer on graphene platelet creates more π bonds. This lowers the energy and increase Raman cross-section. Hence, G frequency decrease and G band intensity increases. Thus, I_D_/I_G_ ratio decreases. This is similar to decrease of G frequency with increase in number of graphene layers. From the same model, it predicts there will be ~3 additional layers after polymer is attached. The grain size of graphene decreases following the immobilization of AO on the polymer surface. This is evident by the increase in the FWHM of G band. This follows the Tuinstra-Koenig relationship I_D_/I_G_ inversely proportional to *L*_a_, where *L*_a_ is the grain size. Hence, it has been found that both G position and I_D_/I_G_ ratio increases. After reacting with AA, it creates a topological disorder in the graphene layer resulting in a loss of some of the aromatic rings for interaction. This weakens the non-covalent bonds and thereby I_G_ increases and I_D_/I_G_ decreases. After reacting with serum and AA, it leads to more amorphization of the graphene layer. The *sp*^*3*^ content of the system increases, which leads to increase of G frequency and decrease of I_D_/I_G_ ratio.

The interaction of AA with AO induces a charge transfer which leads to generation of carriers, and hence, to a modification of conductivity of the system. The enzymatic oxidation of ascorbic acid to dehydroascorbic acid in the presence of ascorbate oxidase produces two electrons, which will be transported through the conductive graphene oxide path to the electronic circuit to register the conductivity change (Fig. 3F). The enzyme ascorbate oxidase (AO), multi-copper enzyme, is chemically proteinase in nature with three various coordination sites^30^. In step one, copper of the AO is bonded to two imidazole groups through the nitrogen and sulfur of cysteine. In step two, copper generates bond with two imidazole groups and in step three, histidines are bonded to every copper^31^,^32^,^33^. The reaction mechanism of ascorbate oxidase with ascorbic acid in presence of oxygen is as follows:^34^


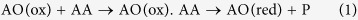



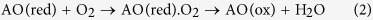


In above two equations, AO (ox) and AO (red) are the oxidized and reduced states of the enzyme; AA is the ascorbic acid (substrate) and P is ascorbate free radical intermediate product. The change in potential can be attributed to the reduction of Cu^+2^ to Cu^+1^ on the enzyme. Because of the accumulated ascorbate ions on the surface of electrode, the electron density around the electrode changes, this in turn is detected by the transducer.

### Analytical performance of the sensor

**Figure 4:**
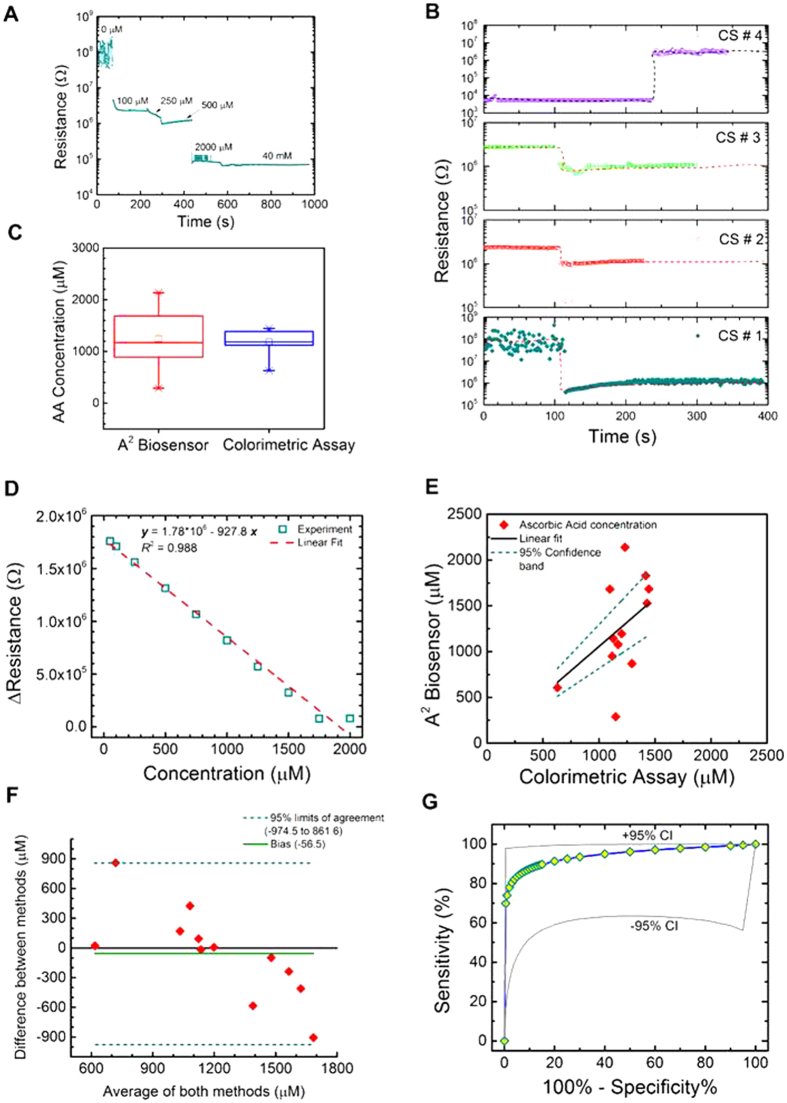
Performance of OcuCheck compared to colorimetric assay measuring ascorbic acid (AA). (**A**) Typical results obtained from OcuCheck showing the concentration dependent resistance measurements. (**B**) Typical resistance measurements obtained from clinical samples on the biosensor. Here, four different clinical samples (labeled CS#1-4) are shown that are measured on four different paper-based biosensors. (**C**) Box plot showing the comparison of OcuCheck and colorimetric assay using clinical samples (n = 12) obtained from the aqueous humor of the eye. (**D**) Calibration curve of OcuCheck using standard AA solution showing the linearity of the biosensor. (**E**) Comparison between OcuCheck and colorimetric assay using clinical sample (n = 12). The dashed lines represent the upper and lower 95% confidence interval (CI). The solid line is a linear fit to the data with the y intercept at 0. (**F**) Bland-Altman plot of results comparing the two methods. The dashed lines represent the upper and lower 95% confidence interval for the level of agreement. The solid cyan curve represents a bias and the solid black line is the line of equality. (**G**) ROC curve along with the 95% CI curve is provided for the OcuCheck.

A calibration curve was generated by measuring the change of resistance of the OcuCheck with various known concentrations of AA placed in a standard solution of 0–40 mM concentration. Experiments were performed using AA standards with concentration of 0–40 mM. At low concentration of AA (<50 μM), the signal-to-noise ratio were not adequate to distinguish between presence and absence of AA. At 50 μM of AA, clear change of resistance was observed (Fig. S5). Each concentration was tested by dropping 10 μL of solution on the sensor. The resistance of the OcuCheck decreased with the increase in concentration of AA as shown in Fig. 4A. The corresponding calibration equation is plotted in Fig. 4D, where the data follows a linear trend (R^2^ = 0.988). In order to measure the concentration of AA in an unknown sample, the measured resistances corresponding to the sample are compared to that of the calibration curve. Optimization of the OcuCheck was achieved with two different designs (Fig. S6). In one of the designs, a low concentration of graphene platelet was used (Fig. S6A). In the other design, a high concentration of graphene platelet was used (Fig. S6B). The graphene platelet formed a largely unconnected system leading to high surface resistance (>6 MΩ) below the percolation threshold as shown in Fig. 4B (CS#1–3; CS: Clinical Sample). When the concentration of graphene platelet was raised above the percolation threshold, a connected system was formed (Fig. 4B, CS#4) leading to lower surface resistance (<0.2 MΩ). These two systems led to two different behaviors for the graphene platelet-AO assembly and with subsequent interactions of AA. For example, the system shown in Fig. 4B (CS#1–3) leads to a decrease of surface resistance due to the presence of ionic solutions like AA (this results from the formation of conductive channels between the graphene platelet islands). In comparison, the system showed in Fig. 4B (CS#4) leads to an increase of surface resistance due to the product formation of catalyzed reaction between AO and AA. Figure 4C shows the Box-and-Whisker plots of the AA concentration from samples of human aqueous humor obtained from subjects who underwent therapeutic anterior chamber paracentesis (n = 12) using OcuCheck and standard colorimetric assay (see the Methods section for the process of aqueous humor collection). The boxes show median and quartiles, where whiskers are corresponding to 5^th^ and 95^th^ percentiles. Levene’s test was performed to quantify the homogeneity of variance. The results (F = 9.45, P = 0.0055) showed that the population variance are significantly different at 0.05 level (also shown in Fig. 4C). One way ANOVA testing of the population of OcuCheck and colorimetric assay (n = 12) showed that (F = 0.113, P = 0.74), at 0.05 level the population means were not significantly different. Tukey test also confirmed the above finding indicating that the difference of means between the OcuCheck and colorimetric assay are not significantly different at 0.05 level. Finally, the t-test (P = 0.678) showed no statistical difference exist between the mean obtained from two methods.

### OcuCheck comparison to colorimetric assay

The validation of the results for the aqueous humor clinical samples was performed by obtaining the concentration through standard colorimetric assay and comparing them to the mass spectrometric based analytical methods. The AA concentration was first determined by a coupled enzyme reaction (AA Assay Kit, Sigma-Aldrich, MAK074), which results in a colorimetric (λ_abs_ = 570 nm)/fluorometric (λ_ex_ = 535/λ_em_ = 587 nm) product, which was proportional to the AA present. Total volume of 200 μL (10 μL of AA, 50 μL of master mix, 140 μL of buffer) with AA concentration ranging from 0–10 nmol/well was used to generate the standard curve. (see Materials and Methods section for sample collection procedure). To measure the concentration of AA in the clinical samples, 10 μL of clinical sample was added to the master mix and buffer (200 μL) to obtain the absorbance/fluorescence data. The concentration of AA was obtained by comparing the absorbance or fluorescence data to the calibration curve generated for the AA standard solution. The comparison between the results obtained from two methods is shown in Fig. 4E. The regression analysis showed strong agreement between the two methods (*R*^2^ = 0.89, Pearson’s *R* = 0.95).

Bland-Altman analysis^35^ was performed to measure the agreement between two quantification methods of AA: colorimetric assay and OcuCheck (Fig. 4F). Bland-Altman plot was obtained by plotting the difference between concentrations measured from two methods (OcuCheck and colorimetric assay) with the mean of the concentrations measured by the two methods. It generated a bias value of −56.5 μM, which indicates that the OcuCheck under predicted the AA concentration compared to the gold standard colorimetric assay. This may be due to the oxidation of AA during sample handling and experimentation. The limit of agreement (95% confidence interval, CI) was calculated by taking 2 x SD of the difference value. Bland-Altman plot was obtained from 12 samples (n = 12) analyzed on the OcuCheck and the Colorimetric assay with correlation R = 0.7761 (P < 0.01), slope = −1.045 (P < 0.01), and intercept = 1219.3 (P = 0.005). The Pearson coefficient represents the linear relationship between the two methods of concentration measurement from OcuCheck and colorimetric assay. Its value ranges from [−1, 1], with 1 representing the perfect correlation of the two methods. The Pearson R = 0.95 with intercept set at 0. P < 0.01 rejected the null hypothesis that there is no correlation between OcuCheck and colorimetric assay measurements.

Further statistical analysis was performed to construct the Receiver Operating Curve (ROC). The detection of AA using OcuCheck has an accuracy of 81.3%, sensitivity of 88.9% [95% confidence interval (CI), 62–100%] and specificity of 71.4%. ROC shows the area under the curve of 0.94 for AA detection. (Figure 4G) The clinical data were analyzed using 6-point rating scale (defined by numbers 1–6) by comparing the results obtained from OcuCheck, colorimetric assay and high resolution mass spectroscopy (HR-MS). The following category of classification was followed to construct the ROC. 1: Definitely negative [MS(N), Colorimetric(N), OcuCheck(N or low)]; 2-Probably negative [MS(N), Colorimetric(N), OcuCheck(Y or High)]; 3-Possibly negative [MS(N), Colorimetric(Y), OcuCheck(N or Low)]; 4-Possibly positive [MS(Y), Colorimetric(N), OcuCheck(Y or High)]; 5-Probably positive [MS(Y), Colorimetric (Y), OcuCheck(N or Low)]; 6-Definitely positive[MS (Y), Colorimetric(Y), OcuCheck(Y or High)]

### AA confirmation using mass spectrometer

**Figure 5:**
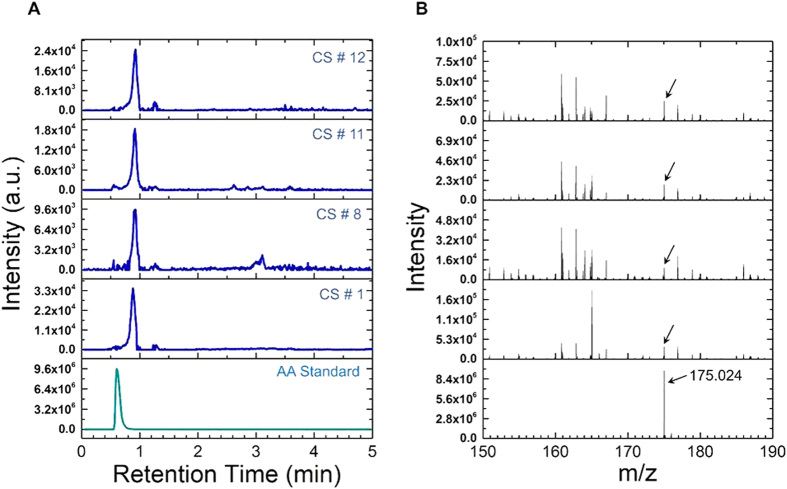
LC chromatogram and HR-MS analysis of clinical samples. (**A**) LC/MS/MS Multiple Reaction Monitoring (MRM) analysis for AA standards and representative clinical samples (CS# 1, 8, 11, 12). (**B**) Corresponding high resolution mass spectrometer (HR-MS) data of AA standard and clinical samples. The characteristic AA fragment is obtained at m/z of 175.024 (denoted by an arrow). The same peak is also seen in all the clinical samples (denoted by arrow) confirming the presence of AA in the samples.

The presence of AA in the clinical sample was confirmed by liquid chromatography (LC) followed by high-resolution mass spectrometry (HR-MS) as shown in Fig. 5A–B. The AA in the clinical sample was converted to a charged (ionized) state, with subsequent analysis of the ions and any fragment ions that are produced during the ionization process, was performed on the basis of their mass to charge ratio (*m*/*z*). The characteristic AA fragment is obtained at *m*/*z* of 175.024 (denoted by an arrow). The same peak is also seen in all the clinical samples (denoted by arrow) confirming the presence of AA in the clinical samples.

## Discussion

Accurate point-of-service diagnostic equipment is needed to improve the delivery of health care. One of the health care areas that can be improved by technology is in the diagnosis of eye disease by primary care physicians. Serious ophthalmic conditions including suspected ruptured globe injuries or post-operative infections require urgent attention and proper management and can first present to primary practioners. Overall eye trauma represents approximately 3% of all emergency medical reports in the United States. The incidence of open globes has been reported to be about 2–6 per 100,000 population in adults^36^ and 15.2 per 100,000 in children^37^. Post-operative infections occur in 14% of specialty glaucoma surgeries called trabeculectomies^38^. It is estimated that 41- 45% of patients who suffer from infection post-trabeculectomy will have severe vision loss of 20/400 or worse^39^,^40^. Many reports stress the importance of vigilance in monitoring post-trabeculectomy patients^41^,^42^. However, no clear consensus exists on how these patients should be followed and early wound leaks, called bleb-leaks, can be missed. In addition, timely diagnosis and early treatment in all of these cases is critical^38^. The lack of an objective test to monitor these conditions is a major unmet medical need in our health care system.

The OcuCheck can replace the subjective Seidel test that is currently the gold standard for evaluating aqueous humor leaks^43^,^44^,^45^,^46^,^47^,^48^,^49^,^50^. This will offer many advantages to the ophthalmology community. We anticipate that the OcuCheck will be a game changer for the evaluation of post-surgical incisions from glaucoma filtering procedures (such as trabeculectomies) as well as for anterior ocular trauma patients.

It has been shown that ascorbic acid concentrations in the tear film are connected to release of the antioxidant from the lacrimal gland with tear film production and do not come from leaking of the molecule through the cornea in normal healthy eyes^51^. We introduce the first use of ascorbic acid as a biomarker for a full thickness corneal injury. We hypothesize that since ascorbic acid is expressed in concentrations that are around five times higher within the aqueous humor on the inside of the eye as compared to within the tear film on the outside of the eye, that a rise in ascorbic acid within the tear films to levels that increases toward the concentration of the aqueous humor would suggest a direct communication. Ascorbic acid has not been studied for use in this role in the past.

In a research sense, the OcuCheck would offer a reliable, objective standard for grading the degree of a wound leak, which could be used to stratify wounds leaks into categories based on severity, with higher severity leaks seen in cases of high AA concentration in the tear film. This may provide researchers with a reproducible way for monitoring post-operative outcomes and could replace alternative methods that are currently used.

Finally, this reliable technology would also be able to revolutionize post-operative management in remote areas or third world countries where access to specialist is limited. In these situations the OcuCheck can be used by health care aids to monitor post-operative patients and used to help in the decision of whether initiation of antibiotics is needed. The OcuCheck will provide critical diagnostic information care providers in order to initiate sight-saving treatments.

One limitation of our study is that we did not measure the AA within human tear film, instead it was tested with aqueous humor samples directly with known higher concentrations of AA. The current proof of concept sensor requires about 2 μL sample volume to get reliable results. In future, we will reduce the sensor area from current 1 cm^2^ to ~few mm^2^ to reduce the sample volume requirement. This is important because the typical tear film turnover rate may be few μL/min. Furthermore, the sensitivity our device makes testing the tear film possible and will be a future study for our device. Also, in order to have a controlled sample of fluid, we did not collect the samples in trauma patients or patients with anterior pathology, instead they were from subjects undergoing treatment for posterior retinal tears. Hence, the biocompatibility of the sensor film has not been tested yet as in the extent that it was design for within this study. Another limitation of our study is the lack of data for long-term stability (greater than 10 weeks) of the sensor devices, and stability of the enzymes at higher temperature (> 37 ^o^C). Also, due to the manual preparation of the sensors, the AO coating is not uniform across the paper-based sensor surface. This contributes to lower sensitivity and specificity. Potentially this can be improved by using machine controlled dispensing system for AO immobilization which will also improve the performance of the device.

In summary, a full-thickness laceration in the cornea or anterior scleral from trauma or incisional surgery releases aqueous humor into the tear film which pathologically increases the concentration of tear film AA to a measurably higher level than that found in normal eyes. This level can be detected with the use of a novel OcuCheck. The results from the Ocucheck can be used as a surrogate biomarker of the integrity of anterior ocular wounds. Current methodologies of absorption and fluorescence based detection are not effective because they have low sensitivity and consume too much time to be clinically relevant in emergency settings. The OcuCheck, which we have developed, offers an electrical resistance measurement based technique that provides an effective and efficient method for testing AA in a POS delivery system. We have shown that the OcuCheck can accurately and quantitatively measure AA levels in *in vitro* testing of aqueous humor samples collected from human subjects. The clinical samples from human aqueous humor were successfully tested to determine the concentration of AA. Complimentary analytical methods such as colorimetric assay and mass spectrometry were utilized to compare and confirm the presence of particular concentration of AA in the clinical samples. The OcuCheck can be refined to make improvements in the specificity and sensitivity in order to use this device in a clinical setting by a vast array of health care providers. The potential impact of this study will be a significant change in the current method for evaluating eye post-surgical patients as well as trauma patients. It will improve the utilization of health care resources and quality of care of patients.

## Materials and Methods

### Study design

The goal of this study was to develop a point-of-service device to measure AA concentration in the human ocular tear film that can be translated to clinical use for evaluation of the anterior of anterior ocular wounds. This research was to establish the proof-of-concept of the device *in vitro* testing. The power analysis suggested that 15 samples would be needed to achieve <5% standard error of mean (SEM) for the OcuCheck biosensor compared to colorimetric test. The aqueous humor AA concentration of 23 μM and standard deviation (SD) of 9.6 μM for healthy individuals are considered for the calculation. The procedure was approved independently by the University of Illinois at Urbana-Champaign (UIUC) and Carle Foundation Hospital Institutional Review Boards (IRBs). All steps of the study upheld the tenants and principles established in the Health Insurance Portability and Accountability Act. During the course of the study, samples of aqueous humor were collected from the Carle Ophthalmology Department and sent to the research laboratory for testing. The samples were collected from patients with localized retinal detachments who were scheduled for therapeutic paracentesis to release intraocular pressure after therapeutic pneumatic retinopexy as standard of care. The fluid was removed in a controlled clinical setting after placement of a topical anesthetic drop of proparacaine and antibiotic drop of ocufloxacin on the cornea of the patient. The fluid was collected using a sterile 30-gauge needle attached to a sterile one-milliliter syringe. After collection, the needle is removed and discarded in the appropriate sharps container for disposal and the syringe containing the fluid sample is then capped and placed in a biohazard specimen collection bag supplied by the hospital. No patient identifiers were connected to the sample. A member of the research team brought the sample to the research laboratory at the Mills Research Center for further testing. The Ophthalmology Department at Carle is directly connected to the research laboratory by the use of a walkway between the buildings. No patient identifiable information was connected to the sample.

### Computational studies

Molecules of AA and sialic acid were sketched and minimized with MOE 2013.08. Force field: MMFF94, Cutoff: 8,10, Dielectric Constant: 1.00. Solvation method: distance model. 1AOZ was subjected to structural preparation before docking. MOE 2013.08 was utilized to perform the docking. Active sites were chosen with the residues Trp163, Trp36μand His512. Induced Fit was chosen as the docking protocol. The Place method was triangle matcher. London dG was used as the scoring method. 30 docking poses were retained and the best scored pose was chosen as the docking pose.

### Preparation of GRP-polymer composites

Graphene platelet (0.1 mg/mL) was mixed with polystyrene-block-polyacrylic acid (PS-*b*-PAA) amphiphilic polymer suspension (0.1 mg/mL) in water. Mixture (GRP-polymer) was probe sonicated at amplitude 4, with pulse of 5 sec on and 1 sec off for 4h with intermittent cycles of 30 min. Process was completed at controlled temperature of 60 °C. Suspension of GRP-polymer was stored at 4 °C in germ free condition before using for preparing GRP-polymer coated filter paper.

### Methodology of surface coating on filter paper

Whatman filter paper of diameter 7 cm was used to be coated with GRP-polymer composites by dip-dry method. Whatman filter paper was dipped in 10 mL of GRP-polymer suspension and incubated at RT for solvent evaporation under controlled atmosphere and deposition of GRP-polymer composite on filter paper. Procedure was performed under biosafety hood on sterilized surfaces and germ free environment.

### Coating of Ascorbate oxidase

To make GRP-polymer coated filter papers specifically responsive to AA, AO was surface loaded to GRP-polymer coated filter papers by drop-cast method in BSL2 facility. Each 1 cm^2^ area of the paper was coated with 12 U of AO which would be enough to interact with 1.2 mg of ascorbic acid specifically. Coated papers were incubated at RT under germ free condition for air drying.

### Formation of interdigitated electrodes

The interdigitated electrodes are fabricated on a filter paper. In order to make electrodes on filter papers without using lithography process, a shadow mask was fabricated on stainless steel substrate. Uniform layer of gold metal with thickness of ~200 nm were deposited through the shadow mask to fabricate the electrodes using Temescal six pocket E-Beam Evaporation System. After the deposition, individual sensors were cut and attached with wire bonding process using silver paste to prepare for further experimentation.

### Characterization of biosensor strips

For atomic force microscopy (AFM) analysis, a square of biosensor strip was attached to AFM disks using double sided carbon tape. AFM images were obtained using an Asylum Cypher (Santa Barbara, CA, USA) with tapping mode and phase mode. The surface of strip was scanned in air using OMCL-AC160TS cantilevers at a set point of 0.63 V, a 1 Hz scan rate, and a drive frequency of 336.3 kHz.

For TEM, a 50 mm diameter section of a biosensor strip was cut from the whole and placed onto the TEM sample holder. Images were obtained using a Jeol (Peabody, MA) 2010 cryo-electron microscope operated at 200 kV, and using different degrees of defocus to obtain an adequate phase contrast. Images were recorded on a Gatan (Pleasanton, CA) UltraScan 2kx2k CCD.

For X-ray color mapping and SEM imaging, a biosensor square was attached to SEM sample holders using two pieces of copper tape to hold the sample in place. Images obtained using a Hitachi (Schaumburg, Illinois) S-4700 SEM with Oxford Instruments (Abingdon, Oxford shire) ISIS EDS X-ray Microanalysis System and Centaurus BSE detector. The biosensor strip was scanned with an accelerating voltage of 10 kV, extracting current of 10 μA, working distance of 12 mm, in analysis mode. X-ray mapping of 70 compiled scans with 250 μs point dwell time and 512 resolutions.

Specificity of the sensor for ascorbic acid using UV-vis spectroscopic studies

An aqueous solution of 60 μg (20 U)/mL ascorbate oxidase was studied for absorbance. Further, aliquots (1 μL; Final concentration 50 μ) of L-ascorbic acid, L-lactic acid and sialic acid (50 mM) were added sequentially till total concentration reached to 1000 μM. Absorption values acquired after total concentration of L-ascorbic acid, L-lactic acid and sialic acid reached 50, 100, 250, 500, 750 and 1000 were plotted against range of wavelength from 200 to 600 nm. Shifts in λ_max_ (x) and absorption efficiency (y) were compared to find out maximum interaction.

### Experimental set-up for resistance measurement

The change in resistance of the graphene platelet-ascorbic oxidase assembly due to presence of different concentration of AA was measured using a digital multimeter connected to a data acquisition system. The data were continuously acquired after dropping the sample on the sensor until the measurement stabilized (about 3 minutes after the start of recording). The stable resistance value was taken for further analysis to plot calibration curve and measurement of concentration of unknown samples.

### Mass spectrometry

AA was bought from Sigma-Aldrich and was used without purification. LC-HRMS was performed on Waters Synapt G2Si Mass Spectrometry. The column model is ACQUITY UPLC BEH C18, 1.7 μm, 2.1 mm x 50 mm. Mobile phase A is 95% H_2_O, 5% CAN, 0.1% FA, and mobile phase B is 5% H_2_O, 95% CAN, 0.1% FA. The linear gradient sequence is 100% A, 0% B (0.5 min), 20% A, 80% B (4.0 min). 100% A, 0% B (4.1 min) with the flow rates at 0.3 mL/min. The auto sampler temperature was at room temperature. Negative mass spectrometry and electrospray ionization (ESI) method was used. 2000 V Voltage was used for ion spray, and the source temperature was 400 °C.

## Additional Information

**How to cite this article**: Gartia, M. R. *et al.* Point-of-service, quantitative analysis of ascorbic acid in aqueous humor for evaluating anterior globe integrity. *Sci. Rep.* **5**, 16011; doi: 10.1038/srep16011 (2015).

## Supplementary Material

Supplementary Information
