# Corrigendum: Point-of-service, quantitative analysis of ascorbic acid in aqueous humor for evaluating anterior globe integrity

**DOI:** 10.1038/srep20452

**Published:** 2016-02-12

**Authors:** Manas R. Gartia, Santosh K. Misra, Mao Ye, Aaron Schwartz-Duval, Lisa Plucinski, Xiangfei Zhou, David Kellner, Leanne T. Labriola, Dipanjan Pan

Scientific Reports 5: Article number: 16011; 10.1038/srep16011 published online: 11032015; updated: 02122016.

The Acknowledgements section in this Article is incomplete.

“SEM images were acquired at Frederick Seitz Materials Research Laboratory Central research facilities, UIUC. Funding: We acknowledge University of Illinois at Urbana-Champaign and Children’s Discovery Institute for financial assistance.”

should read:

“SEM images were acquired at Frederick Seitz Materials Research Laboratory Central research facilities, UIUC. Funding: We acknowledge University of Illinois at Urbana-Champaign and Children’s Discovery Institute for financial assistance. Authors would like to gratefully acknowledge the kind support from Prof. Gang Logan Liu, ECE, UIUC and his laboratory for allowing us to use his inbuilt masking device for deposition of interdigitated gold electrode and impedance based detector for resistance measurement.”

