# Supplementary material for: Point-of-service, quantitative analysis of ascorbic acid in aqueous humor for evaluating anterior globe integrity
Source: Sci Rep. 2015 Nov 3;5:16011. doi: 10.1038/srep16011 (PMC4630616; doi:10.1038/srep16011)
Supplement: Supplementary Information [file srep16011-s1.pdf]

Point-of-service, quantitative analysis of ascorbic acid in aqueous humor for evaluating anterior globe integrity

Manas Ranjan Gartia,[a,d,e,†] *Santosh K. Misra,* [a,d,†] *Mao Ye,* [a,d] *Aaron Schwartz-Duval,*[a,d] *Lisa Plucinski,*[e] *Xiangfei Zhou,*[e] *David Kellner,*[f] *Leanne T. Labriola,*[d,g] *Dipanjan Pan*[a,b,c,d*]

[a] Department of Bioengineering, University of Illinois at Urbana-Champaign

[b] Beckman Institute of Advanced Science and Technology, University of Illinois at Urbana-Champaign

[c] Department of Materials Science and Engineering, University of Illinois at Urbana-Champaign

[d] Carle Foundation Hospital, 611 West Park Street, Urbana, IL, USA

[e] Department of Electrical and Computer Engineering, University of Illinois at Urbana-Champaign

[f] Research Park, University of Illinois at Urbana Champaign, IL

[g] Department of Surgery, University of Illinois College of Medicine, Urbana IL, USA

*To whom correspondence should be addressed: E-mail: dipanjan@illinois.edu

† Equal contribution author.

Supplementary Figures.

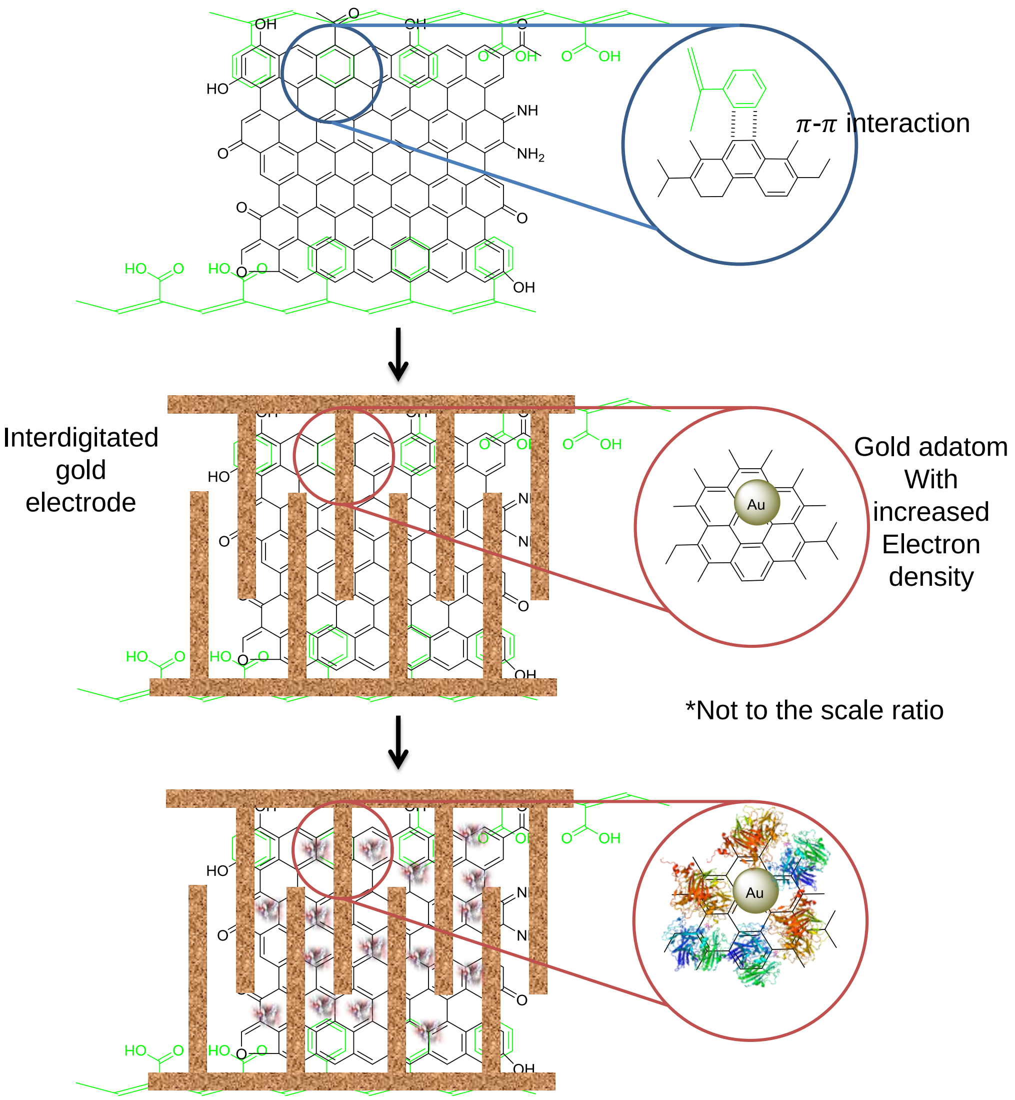

Figure S1 Chemistry of GRP-polymer composites and surface coated filter paper. (A) PS-b-PAA-Graphene platelet composite; (B) interdigitated gold electrode (C) ascorbate oxidase (AO) loaded GRP-polymer coated filter paper; (D) ascorbic acid bound AO.

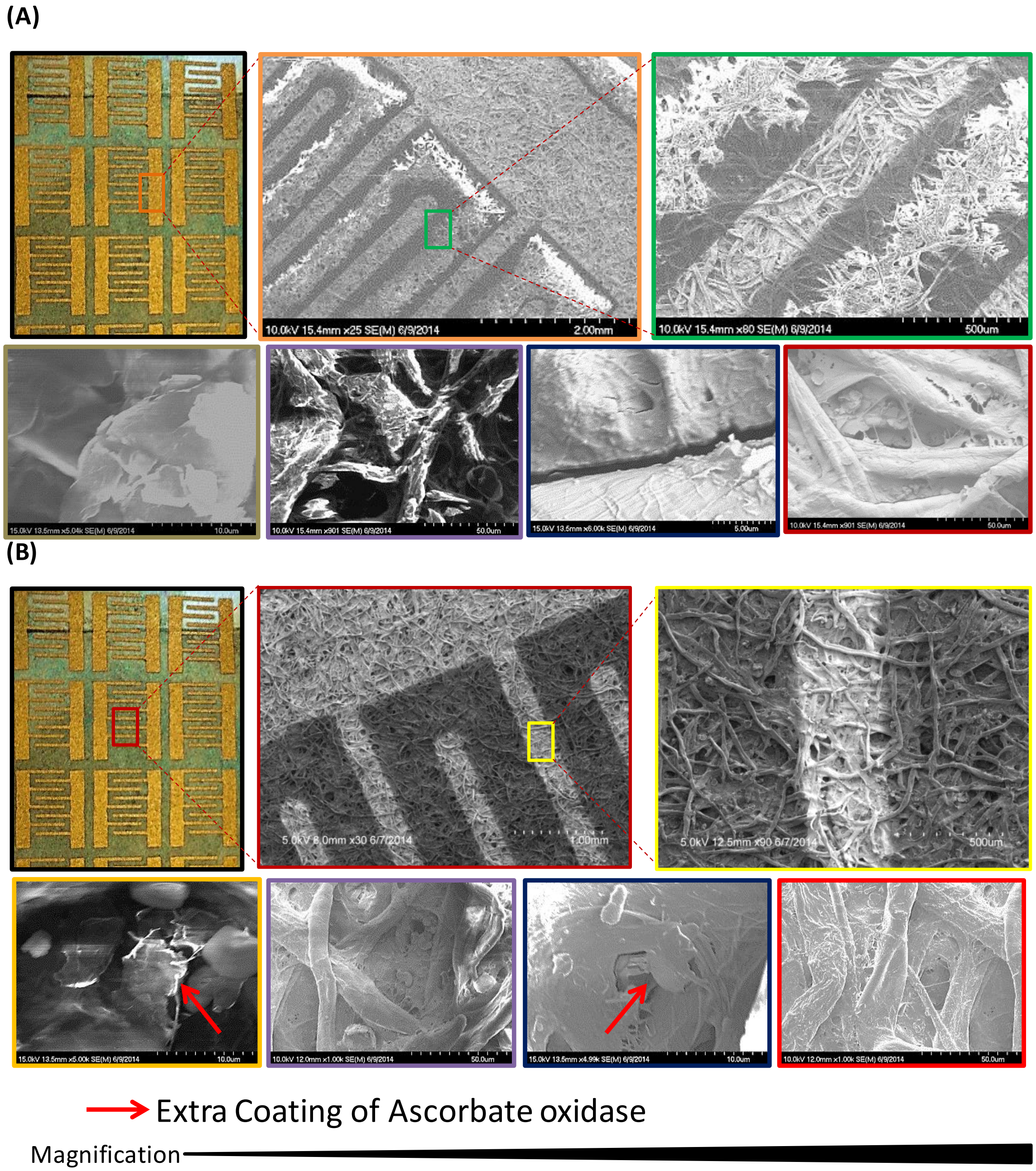

Figure S2 (A) Optical image of OcuChecks and SEM images showing different components without ascorbate oxidase coating and (B) Optical images of OcuChecks and SEM images showing different components with loaded ascorbate oxidase.

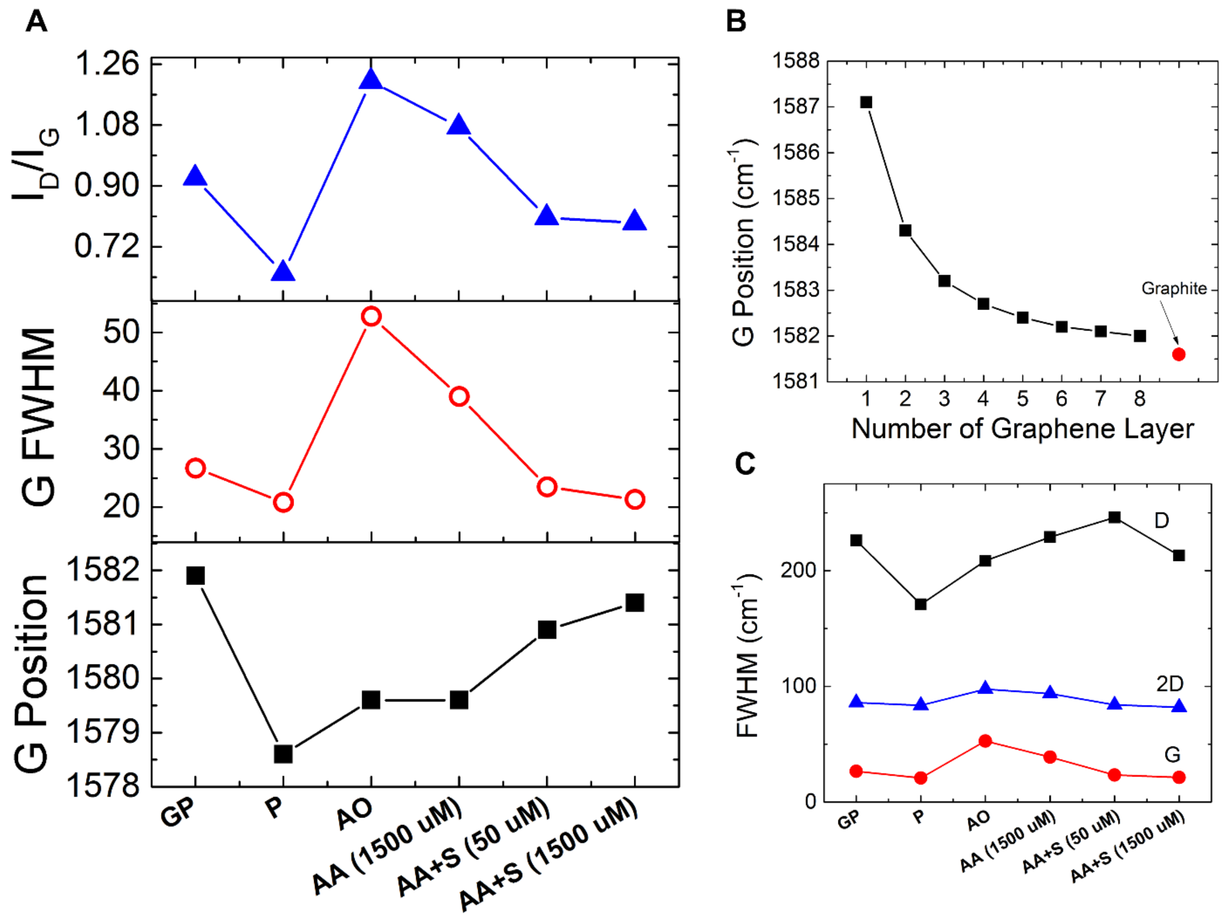

Figure S3 (A) Effect of stacking of polymer, ascorbate oxidase layer and subsequent interaction with ascorbic acid on the G band (position and line width, FWHM) of graphene, and the intensity ratio of D, and G-band of graphene. (B) Results showing the decrease of G-band energy due to increase of number of graphene layers. The G-band frequency can be fitted to the following equation, where n is the number of graphene layer: $\omega_G = 1581.6 + \left(\frac{11}{1+n^{1.6}}\right)$. (C) The effect of stacking of polymer, ascorbate oxidase layer and subsequent interaction with ascorbic acid on the line width of D, 2D, and G-band of graphene.

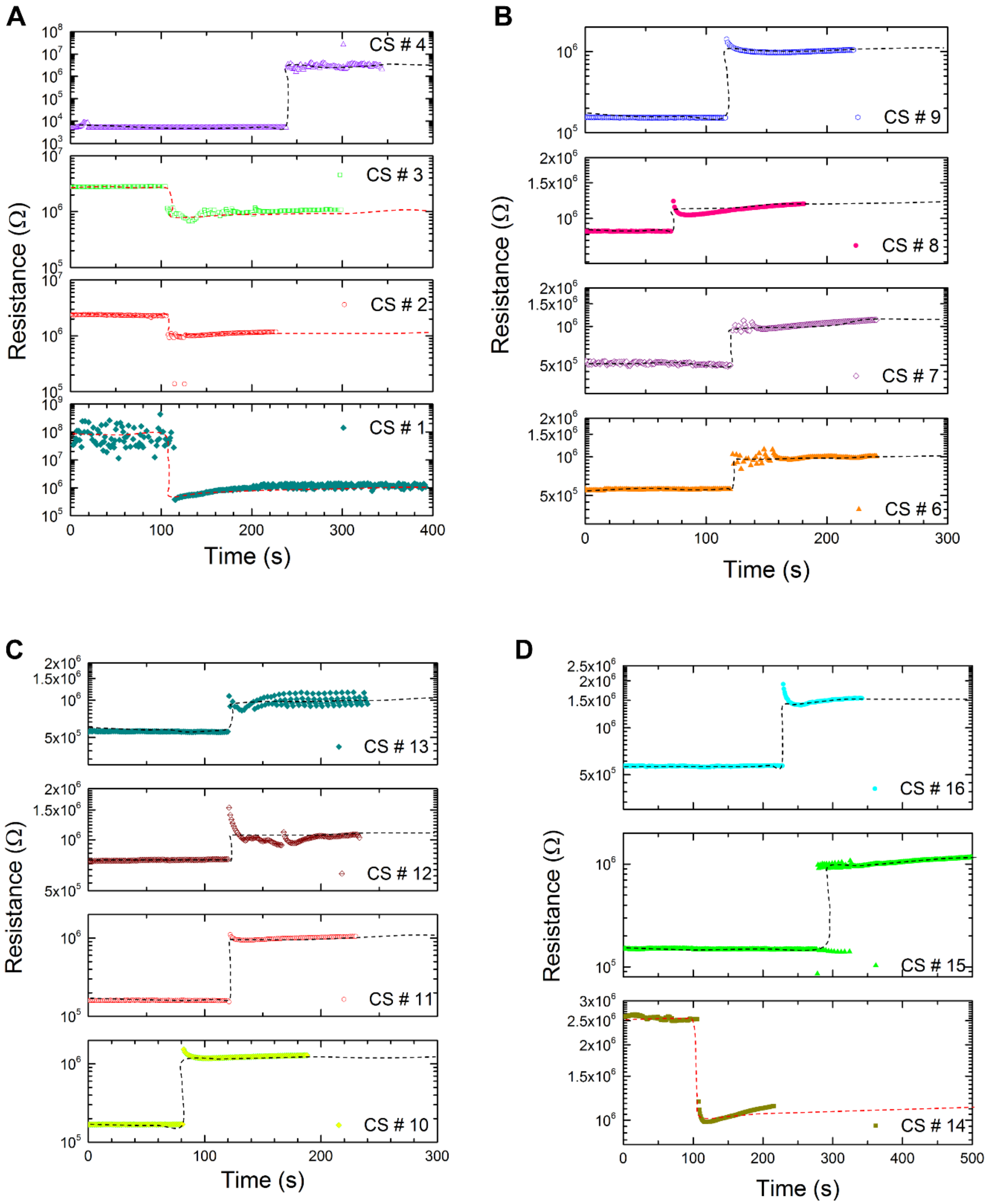

Figure S4 (A-D) Raw data showing results obtained from clinical sample using OcuCheck.

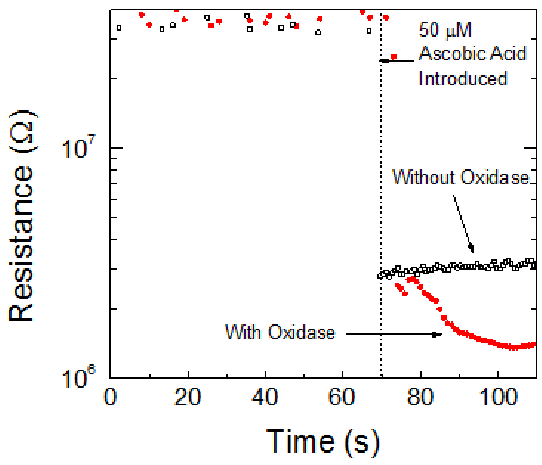

Figure S5. Sensor response at AA concentration of (A) 50 µM and (B) concentration ranging from 0-40,000 µM.

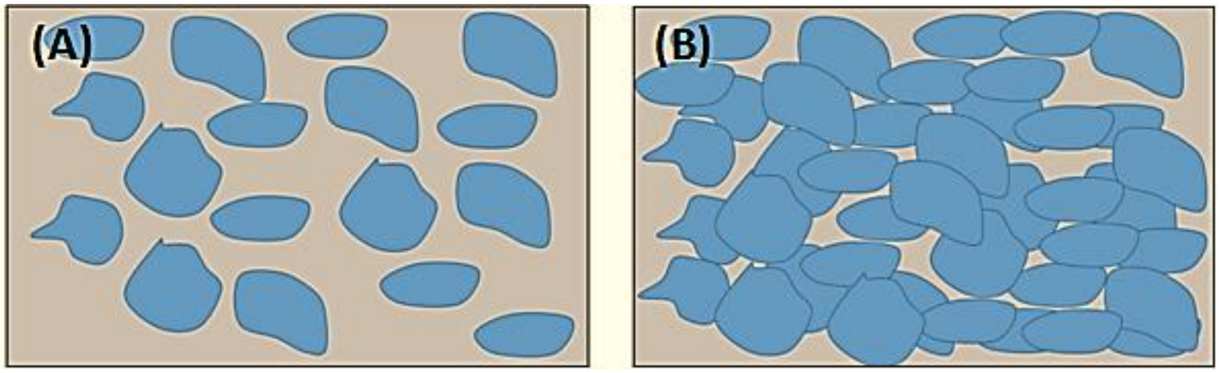

Figure S6. (A) Scenario when the concentration of graphene platelet is low (Type-I). (B) Scenario when the concentration of graphene platelet is high (Type-II).

Table S1. Comparison of various detection methods for their detection limits.

Sl. No.	Method/Transducer	Sample Type	Limit of Detection (nmol/mL)	Reference
1	Resistance change, Graphene platelet	Aqueous humor	50	Present work
2	HPLC-UV	Blood Plasma	0.306	[1]
3	HPLC-UV	Seminal Plasma	10	[2]
4	Electrochemical, screen-printed electrode	AA standards	20	[3]
5	Dissolved oxygen probe	Fruit juice	50	[4]
6	ISFET, MnO_2 nanoparticles	AA standard	10	[5]
7	ISFET, peroxidase	AA standard	500	[6]
8	Impedance, graphite	Fruit juice	2	[7]
9	Potentiometric, ZnO nanorod	AA standard	1	[8]
10	Electrochemical (cyclic voltammetry), Nitrogen doped graphene	AA standard	2.2	[9]

References.

1. Ferin, R., Pavao, M. L. & Baptista, J. Rapid, sensitive and simultaneous determination of ascorbic and uric acids in human plasma by ion-exclusion HPLC-UV, *Clinical Biochemistry*, **46**, 665 (2013).
2. Kandar, R., Drabkova, P. & Hampl, R. The determination of ascorbic acid and uric acid in human seminal plasma using an HPLC with UV detection, *J. of Chromatography B*, **879**, 2834 (2011).
3. Chou, J. C., Tsai, Y. H. & Chen, C. C. Development of a disposable all-solid-state ascorbic acid biosensor and miniaturized reference electrode fabricated on single substrate, *IEEE Sensors J.*, **8**, 1571 (2008).
4. Akyilmaz E. & Dinckaya, E. A new enzyme electrode based on ascorbate oxidase immobilized in gelatin for specific determination of L-ascorbic acid. *Talanta*, **50**, 87–93 (1999).
5. Luo, X.-L., Xu, J.-J., Zhao, W. & Chen, H.-Y. Ascorbic acid sensor based on ion-sensitive field-effect transistor modified with MnO2 nanoparticles. *Anal. Chim. Acta*, **512**, 57–61 (2004).
6. Simonis, A., Dawgul, M., Luth, H. & Schoning, M. J. Miniaturised reference electrodes for field-effect sensors compatible to silicon chip technology. *Electrochim. Acta*, **51**, 930–937 (2005).
7. Veltsistas, P. G., Prodromidis, M. I. & Efstathiou, C. E. All-solidstate potentiometric sensors for ascorbic acid by using a screen-printed compatible solid contact. *Anal. Chim. Acta*, **502**, 15–22 (2004).

8. Ibupoto, Z. H., Usman Ali, S. M., Khun, K. & Willander, M. L-ascorbic acid biosensor based on immobilized enzyme on ZnO nanorods, *J. Biosensors Bioelectronics*, **2**, 3 (2011).
9. Sheng, Z. H., Zheng, X. Q., Xu, J. Y., Bao, W. J., Wang, F. B. & Xia, X. H. Electrochemical sensor based on nitrogen doped graphene: simultaneous determination of ascorbic acid, dopamine and uric acid. *Biosensors and Bioelectronics*, **34**, 125 (2012).
